# Motor Developmental Status of Moderately Low Birth Weight Preterm Infants 

**Published:** 2014-08-15

**Authors:** Azita Tavasoli, Faranak Aliabadi, Rooholah Eftekhari

**Affiliations:** 1 Department of Pediatric Neurology, Ali-Asghar Children’s Hospital; 2 Occupational Therapist, Faculty of Rehabilitation Science, Iran University of Medical Sciences, Tehran, Iran

**Keywords:** Low Birth Weight, Peabody Developmental Motor Scale II (PDMS-2), Motor Development, Infant

## Abstract

***Objective:*** Motor development is frequently reported to be impaired in very low birth weight (VLBW) infants, but little is known about the moderately low birth weight (MLBW) infants. The aim of this study was to investigate whether MLBW preterm infants present developmental delay.

***Methods:*** In a historical cohort study, 18±2 month-old infants with a history of low birth weight (LBW) were identified. All infants with complications of LBW with negative effects on development were excluded. Healthy infants with normal birth weight (2500–4000 g) were included as controls. All infants were evaluated by the Peabody Developmental Motor Scale II (PDMS-2) test and final scores compared between the two groups.

***Finding:*** 88 infants including 58 MLBW and 30 NBW with a mean birth weight of 1900±382.4 g and 3150±473.5 g respectively, were studied. In the MLBW group, gross and fine motor skill scores were below average in 6 (6.8%) and 10 (17%) infants, respectively. There were no significant differences between the two groups according to gross motor quotient (102.5±5.5 in NBW vs 100.1±7.2 in MLBW; *P*=0.1), but MLBW infants achieved significantly lower scores in fine motor (93.3±5.4 vs 99.6±5.0; *P*=0.001) and total motor quotient (97.0±5.9 vs 101.53±5.0; *P*=0.001).

***Conclusion:*** The finding of this study show developmental defects in fine motor skills in MLBW infants. Accurate monitoring of the developmental status of this population should be emphasized for an earlier recognition and intervention.

## Introduction

Low birth weight (LBW) is defined as a birth weight of less than 2500 g and is a major public health problem^[^^1^^]^ as well as a major risk factor for neonatal and postnatal morbidity^[^^2^^]^. Based on WHO statistics the rate of LBW is 17% worldwide (6% in industrialized countries and 21% in developing countries)^[^^3^^]^. Vazirinejad et al found that at a public-sector referral hospital in Iran during a six month period, 9.6% of neonates were LBW^[^^4^^]^. As incidences are substantially higher, the magnitude of the problem is even larger in developing countries^[^^5^^]^. LBW neonates are categorized according to birth weight as follows: 1) moderately low birth weight (MLBW): between 1500 - 2499 g; 2) very low birth weight (VLBW): less than 1500 g; and extremely low birth weight (ELBW) less than 1000 g^[^^6^^]^. The latter groups are at special risk for developmental defects^[^^7^^]^, but MLBW infants may suffer from these problems as well^[^^8^^]^. As the population of MLBW infants is 5 times larger than that of smaller infants^[^^8^^]^, assessment and early detection of motor deficits in order to referring them for interventional program, can lead to the reduction of the later developmental problems and associated costs^[^^9^^]^.

 Recent studies regarding developmental status of LBWs have received limited attention as to the importance of the development of MLBWs^[^^8^^]^. Huddy et al found that these children were at low risk for later neurodevelopmental problems but are prone to academic problems^[^^10^^]^. Middle et al found that MLBW children have higher rates of academic performance and educational problems^[^^11^^]^. Eglan et al and Breslau et al have reported increased rates of learning and behavioral problems among these children^[^^12^^,^^13^^]^. Some of these studies do not include a normal birth weight (NBW) control group for comparison. 

 In this study, we evaluated the motor developmental status of a group of MLBW preterm infants at the corrected age of 18±2 months via the Peabody Developmental Motor Scale II (PDMS-2) test and compared the results with that of NBW infants.

## Subjects and Methods

Upon approval of the ethical committee of human research from Tehran University of Medical Sciences, this study was carried out at Shahid Akbar-Abadi Hospital in Tehran. The sample size was calculated by computing the average and standard deviation of PDMS-2 scores for 20 children (10 LBW and 10 NBW). A total of 52 patients were required to achieve a statistical power of 0.90 with a type I error of 0.05 in consideration of the standard deviation of 11.918. 

 After reviewing hospital records, infants with a corrected age of 18±2 months with a history of preterm birth and MLBWs, from June–November 2008, were included. Infants with birth weights between 1500–2499 g were considered MLBW and with a gestational age of less than 37 weeks were considered preterm. Term infants (with a gestational age ≥37 weeks) with LBW due to intra uterine growth retardation or being small for gestational age, were excluded. In addition, multiple pregnancies, infants with low Apgar scores or severe asphyxia, abnormal brain imaging, congenital malformations, chromosomal and genetic syndromes, and children with history of rehabilitation therapy for more than 2 months or with drug treatments that affected motor function and those who were not residents of Tehran were also excluded. We contacted parents and requested to bring their infant to our clinic and asked for informed consent for the study. The control group consisted of healthy 18±2 month-old infants with birth weights between 2500–4000 g who presented at the clinic for routine checkup. Children with a history of admission to NICU, gestational age of <37 or >42 weeks, resulting from multiple pregnancy, those with musculoskeletal, neurologic, genetic, or any other disorder that negatively influenced development were also excluded. Children in the control group indicated normal development in previous well-child visits by physicians. All parents signed a consent form prior to enrollment in the study. 

 All children were referred to an occupational therapist that was blinded to their birth weight. Their gross and fine motor skills were assessed by the PDMS-2 test, i.e., a specifically designed motor scale that identifies most motor skill dysfunctions. It is a standardized and norm-referenced test of gross and fine motor skills from birth to 5 years of age with a determined reliability and validity^[^^14^^]^. It consists of a Gross Motor and a Fine Motor Scale, each is divided into skill subtests that detect typical motor tasks for each age. Gross motor development is the ability to use the large muscle systems to react to environmental changes, assume a stable posture, move from place to place, and catch, throw, and kick balls. While fine motor development is the ability to use fingers and hands to gasp objects, stack blocks, draw figures, and manipulate objects. Test item performance is summarized and analyzed using motor quotients derived by adding the subtest standard scores and converting the sum to a quotient that has a mean of 100 and a standard deviation of 15. They include gross motor quotient (reflexes or object manipulation, stationary and locomotion subtests), Fine motor quotient (FMQ, gasping and visual-motor integration subtests), and total motor quotient (TMQ) that is comprised of the quotient scores of the gross and fine motor. The total motor quotient (GMQ) is probably the best estimate of overall motor abilities.

 Quotient scores are interpreted as follows: very superior (131–165), superior (121–130), above average (110–120), average (90–109), below average (80–89), poor (70–79), and very poor (35–69).

 Evaluation of the reliability of the tests was performed in a test-retest pilot study. Two occupational therapists independently performed the tests in 10 randomly selected normal children. All children were re-examined by both testers at an interval of one-week. High inter-tester reliability was achieved, as intra-class correlation coefficients (ICC) were 1.00, 0.97, and 0.99 for GMQ, FMQ, and TMQ, respectively (*P*<0.001). The inter-tester reliability also was confirmed. ICCs were 0.94, 0.97, and 1.00 for GMQ, FMQ, and TMQ, respectively (*P*<0.001). The test is an objective assessment tool that does not compromise the results of the study. Nevertheless, we have validated it for the population under investigation that exists as unpublished data. 

 Corrected age for LBW children was calculated by subtracting the gestational age from 37 weeks and the result was subtracted from chronological age (37 -gestational age=A, corrected age=chronological age -A). 

 Statistical analysis was performed using SPSS 17.0 software. Normal distribution of data was tested using the Kolmogorov–Smirnov test. Categorical data was analyzed using the Chi-square test. Continuous variables were compared between the two groups using independent sample t-tests or Mann–Whitney U tests when the distribution of data was abnormal. *P*. value <0.05 was considered significant.

## Findings

A total of 88 infants including 58 LBW and 30 NBW with a mean birth weight of 1900 (±382.4) and 3150 (±473.5) g respectively, were studied. 14 (46.7%) infants in NBW and 30 (51.7%) in LBW group were males, which was not statistically different (*P*=0.6). In the LBW group, there was no significant difference between the mean birth weights of male and female children (1970±423.6 vs 1820 (±430.2) g, respectively; *P*=0.2). The mean age of children in LBW was 18.2 (±0.0) and in NBW 18.0±0.7 months. 

 The mean gestational age of male children in the LBW group was 33.5±2.7 weeks vs 32.3±2.7 weeks in female children (*P*=0.08). The mean duration of hospital stay in LBW group was 7.5 (4–11 days). 

Table 1 demonstrates a comparison of PDMS-2 subtests standard scores for each group. As the data shows, LBW children achieved significantly lower scores in grasping and visual-motor integration skills. There was no significant difference found for stationary, locomotion, and object manipulation skills scores between LBW and NBW children. There was no statistically significant association between gender and motor quotients scores in LBW group (Table 2) and between LBW and NBW groups. According to motor quotients, in the LBW group, GMQ and FMQ were below average in 6 (10.3%) and 10 (17%) children, respectively (Table 2). Table 3 shows that there are no statistically significant difference between groups regarding GMQ (*P*=0.1). However, LBW children achieved significantly lower scores in FMQ (*P*=0.001) and in TMQ (*P*=0.001).

## Discussion

LBW is a major public health problem that negatively influences infant development and the quality of life, and poses financial burdens on health care systems^[^^15^^]^.

**Table 1:** Comparison of Peabody Developmental Motor Scale II (PDMS-2) subtests standard scores in low and normal birth weight children

**Variable**	**Low birth weight** **Mean (SD)**	**Normal birth weight** **Mean (SD)**	***P*** **-value**
**Stationary**	10.5 (1.5)	10.6 (1.3)	0.84
**Locomotion**	9.9 (1.4)	10.4 (1.0)	0.11
**Object Manipulation**	9.6 (1.5)	10.1 (0.9)	0.09
**Grasping**	9.05 (1.0)	9.9 (0.9)	0.001
**Visual-Motor Integration**	8.7 (1.2)	10 (1.0)	0.001

SD: Standard Deviation

**Table 2:** Distribution of motor quotients scores of low birth weight children based on gender

**Parameter**		**Below average (80–89)**	**Average (90–109)**	**Above average (110–120)**
**Gross Motor ** **Quotient**	**Male**	4 (13.3%)	21 (70.0%)	5 (16.7%)
	**Female**	2 (7.1%)	24 (85.7%)	2 (7.1%)
	**Total**	6 (10.3%)	45 (77.6%)	7 (12.1%)
**Fine Motor ** **Quotient**	**Male**	8 (26.7%)	22 (73.3%)	0 (0%)
	**Female**	2 (7.1%)	25 (89.3%)	1 (3.6%)
	**Total**	10 (17.2%)	47 (81.0%)	1 (1.7%)
**Total Motor ** **Quotient**	**Male**	6 (20.0%)	23 (76.7%)	1 (3.3%)
	**Female**	4 (14.3%)	24 (85.7%)	0 (0%)
	**Total**	10 (17.2%)	47 (81.0%)	1 (1.7%)

Several studies have shown that LBW children are more likely to have neurological problems^[^^16^^-^^18^^]^ that may persist into school age and adolescence periods^[^^17^^]^. Motor deficits in LBW children can influence the ability to learn and limit active participation in daily life at school and at home^[^^19^^]^. 

 Our findings indicate that LBW preterm infants at 18±2 month-old corrected age have impaired motor abilities compared with NBW infants especially for fine motor skills. This is in agreement with the results obtained by Cristian Alves da Silva et al in Brazil, who found that LBW preterm infants have delays in neuropsychomotor development and the lowest scores are for language, and hand-eye and fine motor coordination^[^^9^^]^. Our results are also comparable with Goyen T-A, who showed a significant proportion of LBW infants had fine motor deficits at 18 months of age that continued until 5 years of age^[^^19^^]^. Halpern et al reported that LBW children had a three times greater risk of developmental delay compared with NBW (*P*<0.001)^[^^20^^]^. Halpern et al also found the prevalence of infants with delay diminishes when incomes and birth weights increase^[^^21^^]^. In that study, children of poorer families were more prone to developmental delay and birth weight was a strong factor. In addition, Wilcox concluded that low birth weight is strongly associated with later developmental deficits^[^^22^^]^. 

 We did not find any significant difference between LBW and NBW infants regarding gross motor ability. Other studies have shown that developmental problems of LBW children range from mild deficits in cognition and neuromotor functioning in the majority to cerebral palsy in a small minority^[^^2^^,^^16^^,^^17^^]^. 

 We compared motor development between MLBW and NBW infants. Datar and Jacknowitz compared mental and motor development of VLBW and MLBW infants during the first two years of life with NBW, LBWs had a small defect in mental and motor development^[^^23^^]^. Middle et al also reported higher rates of neuro-motor problems in MLBW when compared with NBW children^[^^11^^]^. There were differences between LBW and NBW infants for FMQ and TMQ but not for GMQ in our study. This discrepancy may be related to the visual-motor integration subtest. Goyen et al showed a significant correlation between visual-motor and fine motor skills and concluded that previous reports of visual-motor problems in school-age VLBW children could be due to fine motor defects^[^^19^^]^. Differences observed for TMQ in our study could be a consequence of the magnitude of FMQ. 

**Table 3:** Comparison of motor quotient scores of low and normal birth weight children.

**Parameter**	**Group**	**Number**	**Standard Score ** **(Mean±SD)**	***P. *** **value**
**Gross Motor Quotient**	NBW	30	102.47 (5.52)	0.121
	LBW	58	100.12 (7.18)	
**Fine Motor Quotient**	NBW	30	99.6 (5.02)	0.001
	LBW	58	93.33 (5.42)	
**Total Motor Quotient**	NBW	30	101.53 (5.03)	0.001
	LBW	58	97.02 (5.89)	

NBW: Normal Birth Weight, LBW: Low Birth Weight, SD: Standard Deviation

 Our results showed no differences in motor skills of LBW children regarding gender, which is consistent with those of other studies^[^^3^^,^^24^^,^^25^^]^.

 It has been suggested that early intervention for children who are suspected of motor developmental delay can positively influence the outcome. In a systematic review of 34 studies, Blauw-Hospers and Hadders-Alga have evaluated the effect of intervention from birth to 18 months on outcomes for children who were at increased risk for developmental motor disorders and showed that infants benefit from intervention program^[^^26^^]^. Other studies have shown persistent deficits in LBW children with negative effect on academic performance^[^^9^^]^. Providing early intervention program is a reasonable goal to reduce motor deficit, its consequences, and to improve outcomes. As the problems of VLBW children are more significant and most recent studies have concentrated on this group, physicians may neglect the importance of developmental outcomes for MLBW children. The findings of our study are particularly important because they point to the need to assess the motor developmental status of MLBW infants with follow-ups throughout childhood. 

 Our study has some limitations. First, we have included 18±2 month-old toddlers and therefore, this study cannot answer whether LBW children with delayed fine motor abilities are able to catch up with peers. Second, we used only the PDMS-2 test to assess motor abilities. Although the PDMS-2 is a valid scoring system that is extensively used, some authors have suggested some limitations for it^[^^27^^]^.

## Conclusion

MLBW infants may be at risk for developmental delay, which makes developmental assessments mandatory at an early age for this population. Physicians should take extra care with these infants and proceed to a thorough and systematic monitoring of developmental status. Together with frequent visits, these procedures enhance the developmental delay diagnosis and, hence, lead to earlier recognition and intervention that may reduce long-term problems associated with the developmental delay. 

 Further studies are recommended to investigate motor abilities and the socio-behavioral and cognitive function in late childhood and adolescence of LBW children and to detect the effect of environmental factors on their developmental status.
